# Gallbladder cancer integrated bioinformatics analysis of protein profile data

**Published:** 2019

**Authors:** Mohammad Reza Zali, Mona Zamanian Azodi, Zahra Razzaghi, Mohammad Hossain Heydari

**Affiliations:** 1 *Gastroenterology and Liver Diseases Research Center, Research Institute for Gastroenterology and Liver Diseases, Shahid Beheshti University of Medical Sciences, Tehran, Iran*; 2 *Proteomics Research Center, Shahid Beheshti University of Medical Sciences, Tehran, Iran*; 3 *Laser Application in Medical Sciences Research Center, Shahid Beheshti University of Medical Sciences, Tehran, Iran*; 4 *Proteomics Research Center, faculty of paramedical sciences, Shahid Beheshti University of Medical Sciences, Tehran, Iran*

**Keywords:** Gallbladder cancer, Protein-protein interaction network analysis, Hub-bottleneck proteins, Biological process

## Abstract

**Aim:**

Identifying the critical genes that differentiate gall bladder cancer from a normal gall bladder and the related biological terms was the aim of this study.

**Background:**

The molecular mechanism underlying gall bladder cancer (GBC) trigger and development still requires investigations. Potential therapeutic biomarkers can be identified through protein-protein interaction network prediction of proteome as a complementary study.

**Methods:**

Here, a literature review of proteomics studies of gall bladder cancer from 2010 to 2019 was undertaken to screen differentially expressed proteins in this cancer. A network of 27 differentially expressed proteins (DEPs) via Cytoscape 3.7.1 and its plug-ins was constructed and analyzed.

**Results:**

Ten proteins were introduced as hub-bottlenecks among which four were from DEPs. The gene ontology analysis also indicated that positive regulation of multi-organism process and regulation of response to biotic stimulus are the most disrupted biological processes of GBC considering their relationships with the DEPs.

**Conclusion:**

ACTG, ALB, GGH, and DYNC1H1, and relative biological terms were introduced as drug targets and possible diagnostic biomarkers.

## Introduction

 Introducing valuable biomarkers of gallbladder malignancy as the most known frequent type of the biliary tract cancer is essential for early diagnosis and treatment approaches. This type of cancer is recognized as the fifth frequent malignancy of the gastrointestinal tract (1). While it is a rare type of cancer, its incidence is high in some countries including China, Pakistan, India, and Chili (2-4). The mortality rate of this type of cancer is very high because of its latent phase of trigger and speedy metastatic behavior (5). In this regard, most of the patients diagnosed with this type of cancer are at their advance stage of their disease by the time of diagnosis (5). The first line therapy for this cancer is surgery as other approaches have shown to be ineffective. Nevertheless, surgery is not a suitable method for all patients and only limited numbers of them can benefit from this approach (5). By identifying biomarkers related to the early stage of GC, it is possible to better understand the mechanism of the disease as well as design proper treatments. One of the famous molecular applications for early stage biomarker discovery is proteomics study. Using this approach, a vast number of proteins with differential expression could be detected. These elements are vital for cell biological processes and function. Thus, any dysregulation of these agents could culminate in malfunction of the cell system and consequently abnormal behavior in an organism. The altered expression proteins which are specific and sensitive are known as biomarkers that are valuable for drug targeting (6). However, the biomarkers are not only signal agents that cause the damage; there could be complex interactions between them and other molecules such as genes and metabolites inducing a major disruption in an organism. As such, detection of these connections and the most significant nodes in a system of interaction is worth further examination (7). One of the well-known interactions is called protein-protein interaction network. By analyzing a protein map, it is possible to identify the most key contributors of a network structure and strength as well as the pattern of that specific condition such as a disease (8). Hence, promising biomarkers of a cancer can be evaluated for their interaction properties in a network of PPI connections. In this integrative bioinformatics study, it is aimed to collect the proteomics biomarkers reported for gallbladder cancer and set a complementary analyses of interaction behaviors of these biomarkers, thereby introducing the most key players in this cancer in terms of interaction properties. 

## Methods

The studies of gall bladder cancer proteomics were searched through Google Scholar and PubMed sources. The keywords used for our search were “Proteomics” and “Gall bladder Cancer”. These investigations had been published from 2010 to 2019. The proteins highlighted by these studies were gathered and then chosen for further analysis. Cytoscape v.3.7.1 and String db were used to construct a network of GBC, while the topological features were analyzed by Network Analyzer (9, 10). Note that the parameters for network centrality analysis are degree and betweenness. Nodes with the highest values of degree and betweenness are called hubs and bottlenecks, respectively. Elements with both features are hub-bottlenecks which are the most central proteins of the PPI network. The enrichment analysis of DEPs is the next step which involves a biological process (BP), molecular function (MF), cellular component (CC), and KEGG pathways. Meanwhile, BP has been the designated analysis for differential expressed proteins via ClueGO+Clue Pedia (11, 12). The statistical criteria for this procedure include the minimum number of genes; 2 and percentage in term; 1, respectively. For group P value correction, Bonfferoni step down was used while for enrichment/depletion, two-sided hypergeomtric test was applied. Asterisk signs in the grouping terms indicate statistically significant groups. Two star implies highest significance values of that group, while no star indicates no statistical significance. 

## Results

Table 1 presents a total of 27 DEPs (characterized with about 50% expression change) from investigation of six proteomics which are the utilized samples related to the human gall bladder cancer. 

**Table 1:** The list of DEPs along with Uniprot code and expression condition in GBC

Row	Protein Name	Uniprot Accession	Expression
1	ANXA4(5)	P09525	Up
2	ACTA2 (5)	P62736	Down
3	ALB(5)	P02768	Up
4	Hsp90B(5)	P08238	Down
5	Dync1h1(5)	Q14204	Down
6	ACTG (5)	P63261	Up
7	DES(13)	P17661	Down
8	HTRA1(14)	Q92743	Down
9	TAGLN(14)	P37802	Down
10	CTSZ(14)	Q9UBR2	Up
11	GM2A(14)	P17900	Up
12	CTSH(14)	P09668	UP
13	GGH(14)	Q92820	Up
14	NAGA(14)	P17050	Up
15	NEFH(14)	P12036	Down
16	RSU1(14)	Q15404	Down
17	MUC13(14)	Q9H3R2	Up
18	NUCKS1(14)	Q9H1E3	Up
19	DMBT1(14)	Q9UGM3	Up
20	HMGB2(14)	P26583	Up
21	LAMB3(14)	Q13751	Up
22	PSAP(14, 15)	P07602	Up
23	MIF(15)	P14174	Up
24	ANK3(16)	Q12955	Down
25	FHL1(16)	Q13642	Down
26	ANXA3(17)	P12429	Up
27	S100A8(14)	P05109	Down

**Figure 1:**
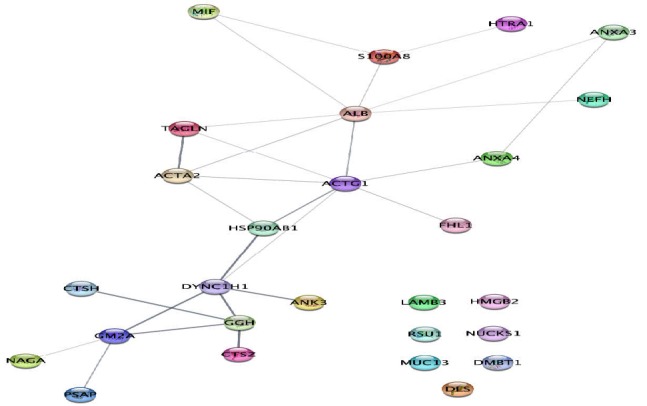
String network of 27 DEPs of GBC with 27 links; Seven proteins remained as individual proteins in the network

Cytoscape via String db interaction analysis of the integrated DEPs based on Table 1 is presented in Figure 1. 

Addition of 50 proteins from STRING database to the main network of 27 DEPs leads to participation of the isolated proteins in the interactome. 

**Figure 2:**
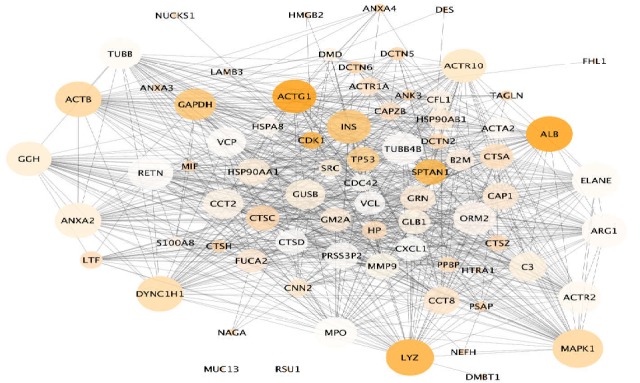
String network of 77 nodes with 917 links; Nodes with the highest degree and betweenness values are larger in size and darker orange, respectively

**Figure 3:**
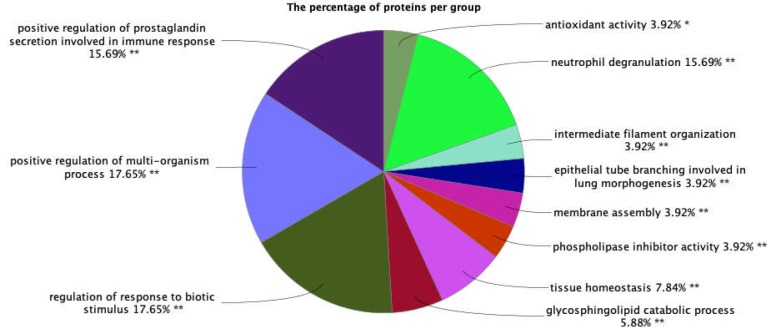
A pie chart view of biological processes identified by ClueGO for 27 DEPs. The astricks indicate the signficance of the grouping

**Table 2:** The hub-bottlenecks of the second network query are ranked based on degree value. The nodes assigned with star are from the query proteins (DEPs)

Row	Display name	Protein name	Degree	BC
1	MAPK1	Mitogen-Activated Protein Kinase 1	43	0.03
2	LYZ	Lysozyme	41	0.04
3	DYNC1H1*	Cytoplasmic dynein 1 heavy chain 1	40	0.02
4	ALB*	Albumin	40	0.05
5	ACTB	Beta-actin	40	0.03
6	ANXA2	Annexin A2	40	0.02
7	GGH*	Gamma-Glutamyl Hydrolase	39	0.02
8	ACTR10	Actin Related Protein 10	39	0.02
9	ACTG*	Actin Gamma 1	38	0.05
10	INS	Insulin	38	0.03

The resultant network with centrality values, analyzed and visualized via Network Analyzer, is shown in Figure 2. 

MUC13 and RSU1 remain as individuals after addition of 50 neighbor proteins to the query proteins. Some nodes show larger centrality values in terms of degree and betweenness, suggesting that the network is scale free. 

Centrality analysis of the second network conducted by the Network Analyzer, the Cytoscape plug-in as the hub-bottlenecks, is tabulated in Table 2. 

According to Table 2, ten hub-bottlenecks of this network contain four DEPs called DYNC1H1, ACTG, GGH, and ALB. The other proteins, on the other hand, could also be important in the GBC pathogenicity. In this regard, literature review of all central proteins has been considered in our study. DYNC1H1, ACTG, and ALB are reported as DEPs of GBC by a proteomics approach (5). MAPK1 as the most highlighted hub is a key differential protein in transition from GBC non-invasive to invasion condition based on a proteomics study (18). ALB and ACTG have the highest BC and as mentioned they are also from DEPs. 

Gene ontology analysis via ClueGO has been conducted for DEPs in terms of biological process identifications. The pie chart summarizes the terms in groups of BPs. Single asterisk (*) shows p<0.05 and Double asterisk (**) implies p<0.01. Groups without stars are not statistically significant which have been omitted in this analysis by setting the query to show only groups with P value less than 0.05, see Figure 3. 

Eleven groups have been identified using ClueGO, among which one has one star suggesting it as significantly lower than 0.01. Positive regulation of multi-organism process and regulation of response to biotic stimulus are the most highlighted groups in the BP query of DEPs based on the number of gene participations. These two groups claim around 18% of all groups in terms of protein participations. Both of these groups are statistically highly significant based on the Group P value Corrected with Bonferroni step down as 0.005 and 0.00002. The second ranked groups in this query are positive regulation of prostaglandin secretion involved in immune response and neutrophil degranulation claiming 16% of all groups. 

The list of proteins participating in the first and second groups include CTSH, DMBT1, HMGB2, MIF, MUC13, NUCKS1, PSAP, S100A8, HTR1A1, and CTSH, DMBT1, HMGB2, HTRA1, MIF, MUC13, PSAP, S100A8 , respectively. The third and fourth groups also consist of proteins including MIF, CTSH, S100A8, MUC13, HTRA1, PSAP, DMBT1, HMGB2, and MIF, S100A8, CTSZ, CTSH, GGH, PSAP, GM2A, ANXA3, respectively. On the other hand, some proteins reveal more associations with the bp groups than with the others. These proteins are CTSH, PSAP, and S100A8 where the first two are linked to six groups while the latter one is associated with five groups.

## Discussion

GBC is the most fatal fast growing type of cancer of the biliary tract (19) where only 20% of these patients are diagnosed as un-metastatic (20). Molecular investigation could provide essential knowledge of this lethal cancer especially in terms of protein dysregulations and their crucial interactions. There are some proteomics studies available from 2010 to 2019 which identified many key proteins. We performed a meta-analysis of the 27 DEPs introduced by these studies via bioinformatics. Different patterns of expressions are assigned for these proteins in the GBC. Taken together, these proteins could provide insight into molecular mechanisms of GBC and more precisely via network analysis. A network analysis of DEPs showed that seven proteins including MUC13, NUCKS1, RSU1, HMGB2, DES, DMBT1, and LAMB3 remained as individual proteins in the first query. As we added 50 neighbor proteins to the query proteins, RSU1 and MUC13 remained as separate proteins. Thus, the rest of individual query proteins from the first network joined the main network of interactions. This demonstrates that these two latest mentioned proteins do not show definite interactions with the rest of DEPs. 

As the analysis continued with the centrality identification of GBC, hub-bottlenecks, i.e. key proteins were introduced. Among the ten hub-bottlenecks, four proteins belonged to the differential expressed set of GBC. These proteins were DYNC1H1, ACTG, GGH, and ALB with the first one being down-regulated while the rest up-regulated in GBC. It can be inferred that up-regulation is dominant among the DE hub-bottlenecks in this study. 

Although MAPK1 does not belong to the list of DEPs in GBC, its phosphorylation has been mentioned in one proteomics study (18). This protein has been additionally reported for other types of tumors such as cervical, colon, as well as head and neck cancers (21-24). In addition, some relationships between MAPK1 and GBC have been reported by other studies as well promoting the idea that this protein may have a contribution to GBC initiation likewise (25, 26). However, additional examinations are required in this regard. 

Lysozyme, the next ranked hub-bottleneck of the GBC network, has been reported with high expression in the sera of patients with malignancies including cancers of lung, melanoma (27), and breast carcinomas (28). This protein also shows metaplastic alterations in gallbladder cancer developments (29). DYNC1H1 is the third ranked hub-bottleneck and as the first ranked in DE hub-bottleneck. This protein indicates down-regulation in GBC and is highlighted as diagnostic biomarker by a proteomics study (5). Further, this protein is important in other cancers as also indicated by literature reviews (30). This protein has many fundamental responsibilities in a cell one of which is contribution to miotic process which plays a key role in cancer (31). Albumin the next ranked hub-bottleneck and second ranked as DE hub-bottleneck is up-regulated in GBC according to the same proteomics evaluation (5). ACTB, the beta actin, has some linkage to different types of tumors. This house-keeping element has mostly showed over-expression in different cancers (32). Regarding GBC, it is still to be investigated and might have some connections. The sixth ranked hub-bottleneck, ANXA2 regulation changes is pinpointed as high expression in GBC by one immunohistochemical research (33). In addition, it is accounted as a trustworthy marker as an approach to screening and treatment follow-ups of many kinds of cancers (34, 35). Gamma-Glutamyl Hydrolase, the third ranked DE hub-bottleneck, is up-regulated in GBC as mentioned by a proteomics investigation (14). It is also highly expressed in other types of cancers as well (36-38). Considering ACTR10, the eighth ranked hub-bottleneck, there is no report concerning its relationship with either with gall bladder cancer or with any other cancer types. ACTG the ninth ranked hub-bottleneck and fourth among DE hub-bottlenecks, showed up-regulation in GBC assigned with a proteomics study (5). Its over-expression has been previously reported for skin cancer (39) as well. The crucial role of gamma actin in cancer is mitotic process and centrosome performance regulations (40). Finally, the last important hub-bottleneck of the network is INS, insulin, which is known as cancer metabolism promoter (41). Collectively, the DE proteins among the hub-bottlenecks, ACTR10 was the only protein not reported for any types of cancers while the rest, based on previous reports showed some connections with different kinds of cancers. Interestingly, MAPK1, ANXA2, and LYZ specified some alterations in GBC by other types of studies rather than proteomics. Thus, they could be important as well for GBC pathogenicity. The next step was to evaluate biological processes related to DEPs. Significant biological processes (p-value <0.01; labeled by ** and p-value, 0.05; labeled by * in the figure 3) were considered. In this way, the aberrant processes of gall bladder cancer could be explored. Indeed, changes in the DE proteins could result in the malfunction of the related biological processes, especially for the DE hub-bottlenecks, it has additional values. As mentioned before, in this investigation, there are some DE proteins based on meta-analysis of proteomics data that indicate centrality values in the network of GBC, including ACTG, ALB, GGH, and DYNC1H1. Hence, the expression changes of these central proteins could conclude in extensive abnormal behavior in our network and accordingly the development of GBC. Further, MAPK1 as the most central hub-bottleneck of the GBC network has previously been found as a DEP in invasion behavior of GBC which could also have a role in other stages of this cancer type that warrants additional analysis in this regard. 

A panel of biomarkers could be more trustworthy than only one assigned biomarker. In this regard, proteins that have been reported by proteomics studies for GBC were gathered and those with greater importance in terms of interactions were introduced as ACTG, ALB, GGH, and DYNC1H1. This panel could be suggested as a promising therapeutic target for screening of GBC once confirmed by complementary evaluations. The follow up of patients and evaluation of treatment are the two important features of application of this finding if more investigation validates our outcomes.
